# *Geastrumsuae* sp. nov. (Geastraceae, Basidiomycota) a new species from Yunnan Province, China

**DOI:** 10.3897/BDJ.11.e99027

**Published:** 2023-03-15

**Authors:** Zheng-Quan Zhang, Chao-Hai Li, Lin Li, Hong-Wei Shen, Jun He, Xi-Jun Su, Zong-Long Luo

**Affiliations:** 1 College of Agriculture and Biological Science, Dali University, Dali, China College of Agriculture and Biological Science, Dali University Dali China; 2 College of Pharmacy, Dali University, Dali, China College of Pharmacy, Dali University Dali China; 3 Center of Excellence in Fungal Research, Mae Fah Luang University, Chiang Rai, Thailand Center of Excellence in Fungal Research, Mae Fah Luang University Chiang Rai Thailand; 4 School of Science, Mae Fah Luang University, Chiang Rai, Thailand School of Science, Mae Fah Luang University Chiang Rai Thailand; 5 Biotechnology and Germplasm Resources Institute, Yunnan Academy of Agricultural Sciences, Kunming, China Biotechnology and Germplasm Resources Institute, Yunnan Academy of Agricultural Sciences Kunming China

**Keywords:** Geastraceae, ITS, nrLSU, taxonomy, phylogeny

## Abstract

**Background:**

*Geastrum* is the largest genus of Geastraceae and is widely distributed all over the world. Four specimens which belong to *Geastrum* were collected during our scientific expedition to Cangshan Mountain, Yunnan, China. Based on morphological characteristics and phylogenetic analysis, a new species was introduced.

**New information:**

*Geastrumsuae* is characterised by its large basidiomata (height 35–70 mm, diameter 18–37 mm) with long stipe (height 10–45 mm), smooth pink exoperidium and sessile globose endoperidial body. Phylogenetic analysis has been carried out, based on the internal transcribed spacer (ITS) and large subunit ribosomal ribonucleic acid (nrLSU) sequence data. The illustration and description for the new taxa are provided.

## Introduction

*Geastrum* Pers. is the largest genus of Geastraceae and was established by Persoon (1794). *Geastrum* is commonly known as the earthstars with worldwide distribution and the most species-diverse in the family Geastraceae. Up to now, there are 140 valid species in this genus (Wijayawardene et al. 2022, Zhou et al. 2022, Cabral et al. 2022, Wang and Bau 2023). *Geastrum* clearly differs from *Myriostoma* by a single endodermal stoma (Sousa et al. 2014). Due to the non-splitting ectoderm and the poorly-developed endoperidium being different from *Geastrum*, researchers thought that *Radiigera* is one of the genera closely related to *Geastrum* (Sunhede 1989, de Toledo and Castellano 1996). Later, some studies have found that specimens in *Radiigera* are nested in *Geastrum* (Hosaka et al. 2006, Hosaka and Castellano 2008, da Silva et al. 2013), but the relationship between these two genera has not been studied in depth until Jeppson et al. (2013) classified the species of *Radiigera* into the genus *Geastrum*. Species of this genus are distributed globally, especially in temperate and tropical regions, such as Brazil-Amazon and Europe (de Leόn 1968, da Silva et al. 2013, Jeppson et al. 2013, Zamora et al. 2014, Cabral et al. 2014a, Cabral et al. 2014b, Sousa et al. 2015, Crous et al. 2016, Cabral et al. 2017, Crous et al. 2017, Sousa et al. 2017, Crous et al. 2018a, Crous et al. 2018b, Assis et al. 2019, Finy et al. 2021, Rodrigues et al. 2021). However, the taxonomic relationship under the genus was chaotic (Zamora et al. 2013) until Zamora et al. (2014) divided it into 14 Sections using polygenic analysis, viz. Sect. Campestria, *Corollina*, *Elegantly*, *Exareolata*, *Fimbriata*, *Fornicata*, *Geastrum*, *Hariotia*, *Hieronymia*, *Myceliostroma*, *Papillata*, *Pseudoilmbata*, *Schmidelia* and *Trichaster*.

In China, the early systematic report of *Geastrum* can be found in "Fungi in China" (Deng 1963) and "The Confluence of Chinese Fungi" (Dai 1979). Zhou et al. (2007) detailed descriptions of 16 species of *Geastrum* in China in "Flora Fungorum Sinicorum-Geastraceae and Nidulariaceae". Later, three new records and nine new species were reported (Han and Bau 2016, Zhou et al. 2022, Wang and Bau 2023).

Four specimens which belong to *Geastrum* were collected during our scientific expedition to Cangshan Mountain, Yunnan, China. Morphological and phylogenetic analysis revealed that these specimens are the same species and are different from other species in *Geastrum*. Therefore, we introduced it as a new species and provided the detailed description and illustration.

## Materials and methods

### Morphological description

Macro-morphological descriptions were based on fresh specimens, which were photographed in the field with notes and laboratory supplemental measurements. The colour is compared with the standard colours in the colorhexa website (https://www.colorhexa.com). Micro-morphological data were obtained from the fresh specimens and observed by using a light microscope, following Accioly et al. (2019). Sections were studied at magnification of up to 1000× using a NiKon eclipse Ni microscope and phase contrast illumination and scanning electron microscope (SEM) analysis was done under a Shimadzu SSX–550. Preparation of the material examined under SEM followed da Silva et al. (2011). Microscopic features and measurements were made from slide preparations stained with 5% potassium hydroxide (KOH). Basidiospore features, hyphal system, colour, sizes and shapes were recorded and photographed. Measurements were made using the Image Framework v.0.9.7 to represent variation in the size of basidiospores, 5% of measurements were excluded from each end of the range and extreme values are given in parentheses.

The abbreviation for spore measurements (n/m/p) denote “n” spores measured from “m” basidiocarps of “p” specimens. Basidiospore dimensions (and “Q” values) are given as (a) b–av–c (d), where “a” represents the minimum, “d’ the largest, “av” the average “b” and “c” covers a minimum of 90% of the values. “Q” is the length/width ratio of a spore inside view and “Qm” for the average of all spores ± standard deviation. Voucher specimens are deposited in the Herbarium of Cryptogams, Kunming Institute of Botany Academia Sinica (KUN-HKAS).

### DNA extraction, PCR amplification and sequencing

The DNA extractions were performed from a small piece of the dried basidioma by using Trelief ^TM^ Plant Genomic DNA Kit from Tsingke Biotechnology Co., Ltd (Beijing, China). Two DNA regions were amplified: the internal transcribed spacer nuclear ribosomal DNA (ITS), nuclear ribosomal large subunit (nrLSU) with the primer pairs ITS1F/ITS4 and LR0R/LR5, respectively (Table 1).

PCR reactions (25 μl) contained mixture: 12.5 μl 2X SanTaq PCR Master Mix (including MgCl_2_, dNTP, Taq DNA Polymerase, PCR buffer, loading etc.), 1 μl each of primer, 2 μl DNA solution and 9.5 μl sterilised distilled H_2_O. The PCR cycling for ITS and nrLSU was as follows: initial denaturation at 94°C for 5 min, followed by 35 cycles at 94°C for 30 sec, 53°C for 30 sec and 72°C for 50 sec and a final extension of 72°C for 10 min. The PCR products were visualised via UV light after electrophoresis on 1% agarose gels stained with ethidium bromide. Successful PCR products were sent to Sangon Biotech Limited Company (Shanghai, China), using forward PCR primers. When sequences have heterozygous INDELS or ambiguous sites, samples were sequenced bidirectionally to make contigs of the amplified regions or verify the ambiguous sites. Raw DNA sequences were assembled and edited in Sequencher 4.1.4 and the assembled DNA sequences were deposited in GenBank (Table 2).

### Sequence alignment

Sequence data of two partial loci, internal transcribed spacer region (ITS) and the large subunit ribosomal RNA gene (nrLSU) were analysed. All the sequences, except those which were obtained from this study, were selected from GenBank for phylogenetic analyses (Table 2). Sequences were aligned using the online version of MAFFT v.7 (http://mafft.cbrc.jp/alignment/server/) (Katoh and Standley 2013) and adjusted using BioEdit v.7.0.9 (Hall 1999) by hand to allow maximum alignment and minimise gaps. Ambiguous regions were excluded from the analyses and gaps were treated as missing data. AliView 1.19-beta was used to convert the alignment fasta file to Phylip and Nexus format for phylogenetic analysis. Phylogenetic analyses were obtained from Maximum Likelihood (ML) and Bayesian Inference (BI).

### Molecular phylogenetic analyses

The Maximum Likelihood (ML) and Bayesian Inference (BI) methods were used to analyse the combined dataset of ITS and nrLSU sequences. ML analysis was conducted with RAxML-HPC2 on the CIPRES Science Gateway (Miller et al. 2010), involving 100 ML searches; all model parameters were estimated by the programme. The ML bootstrap values (ML-BS) were obtained with 1000 rapid bootstrapping replicates.

Bayesian analysis was performed with MrBayes v.3.2 (Ronquist et al. 2012), with the best-fit model of sequence evolution estimated with MrModelTest 2.3 (Nylander et al. 2008) to evaluate posterior probabilities (PP) (Rannala and Yang 1996, Zhaxybayeva and Gogarten 2002) by Markov Chain Monte Carlo (MCMC) sampling. Six simultaneous Markov chains were run for 100,000,000 generations, trees were sampled every 500^th^ generation and 200,000 trees were obtained. The first 50,000 trees, representing the burn-in phase of the analyses, were discarded, while the remaining 150,000 trees were used for calculating posterior probabilities in the majority rule consensus tree (the critical value for the topological convergence diagnostic is 0.01).

The phylogenetic tree was visualised with FigTree version 1.4.0 (Rambaut 2012) and made in Adobe Illustrator CS5 (Adobe Systems Inc., USA). Sequences derived in this study were deposited in GenBank (http://www.ncbi.nlm.nih.gov).

## Taxon treatments

### Geastrum suae

Z.Q. Zhang C.H. Li & Z.L. Luo sp. nov.

0FAB460F-D4E4-5D98-A6C1-E17EBFB315A8

MB845193

#### Materials

- **Type status:** Holotype. **Occurrence:** recordNumber: SJ582; recordedBy: Zheng-Quan Zhang; occurrenceID: 6D676216-9572-5A8D-A7C3-4C445C671395; **Taxon:** scientificName: *Geastrumsuae*; kingdom: Fungi; phylum: Basidiomycota; class: Agaricomycetes; order: Geastrales; family: Geastraceae; genus: Geastrum; **Location:** verbatimElevation: 2160 m; locationRemarks: China, Yunnan Province, Dali City, Cangshan Mountain; verbatimLatitude: 25°43′36.97″N; verbatimLongitude: 100°07′16.46″E; **Event:** year: 2020; month: September; day: 4; habitat: Terrestrial; fieldNotes: grows in groups on the ground in mixed coniferous and broad-leaved forests, with thick humus; **Record Level:** type: KUN-HKAS 123795
- **Type status:** Paratype. **Occurrence:** recordNumber: MB015; recordedBy: Chao-Hai Li; occurrenceID: F58D4D0C-33BB-541B-A92B-7AF436F12F49; **Taxon:** scientificName: *Geastrumsuae*; kingdom: Fungi; phylum: Basidiomycota; class: Agaricomycetes; order: Geastrales; family: Geastraceae; genus: Geastrum; **Location:** verbatimElevation: 2221 m; locationRemarks: China, Yunnan Province, Dali City, Cangshan Mountain; verbatimLatitude: 25°40′16.38″N; verbatimLongitude: 100°09′08.42″E; **Event:** year: 2020; month: October; day: 14; habitat: Terrestrial; fieldNotes: grows in groups on the ground in mixed coniferous and broad-leaved forests, with thick humus; **Record Level:** type: KUN-HKAS 123796
- **Type status:** Other material. **Occurrence:** recordNumber: SJ2501; recordedBy: K. Wang; occurrenceID: 9213A508-19C3-5A9C-8E34-8DCDD9D4C170; **Taxon:** scientificName: *Geastrumsuae*; kingdom: Fungi; phylum: Basidiomycota; class: Agaricomycetes; order: Geastrales; family: Geastraceae; genus: Geastrum; **Location:** verbatimElevation: 2208 m; locationRemarks: China, Yunnan Province, Dali City, Cangshan Mountain; verbatimLatitude: 25°40′28″N; verbatimLongitude: 100°08′59″E; **Event:** year: 2021; month: September; day: 3; habitat: Terrestrial; fieldNotes: grows in groups on the ground in mixed coniferous and broad-leaved forests, with thick humus; **Record Level:** type: KUN-HKAS 123793
- **Type status:** Other material. **Occurrence:** recordNumber: SJ2500; recordedBy: G. H. Yang; occurrenceID: CE65C858-1CD7-53B3-B545-F1750391FB8E; **Taxon:** scientificName: *Geastrumsuae*; kingdom: Fungi; phylum: Basidiomycota; class: Agaricomycetes; order: Geastrales; family: Geastraceae; genus: Geastrum; **Location:** verbatimElevation: 2350 m; locationRemarks: China, Yunnan Province, Yangbi County, Cangshan Mountain; verbatimLatitude: 25°41′59″N; verbatimLongitude: 100°02′00″; **Event:** year: 2021; month: October; day: 1; habitat: Terrestrial; fieldNotes: grows in groups on the ground in mixed coniferous and broad-leaved forests, with thick humus; **Record Level:** type: KUN-HKAS 123794

#### Description

**Figure 1.**
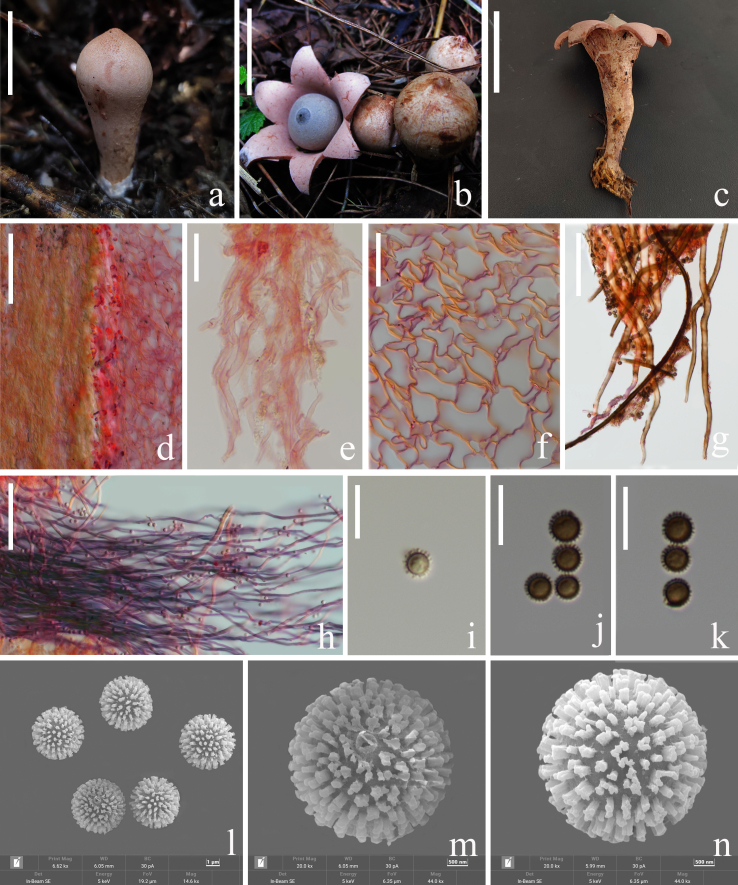
*Geastrumsuae* (KUN-HKAS 123795, holotype). **a** fresh unexpanded fruiting bodies; **b, c** fresh mature fruiting bodies; **d** mycelial layer, fibrous layer and pseudoparenchymatous layer; **e** hyphae of mycelial layer; **f** pseudoparenchymatous layer (cells in the stack); **g, h** eucapillitium hyphae; **i-k** basidiospores (LM); **l-n** basidiospores (SEM). Scale bars: a = 10 mm; b, c, e = 20 mm; d = 80 μm; f, g, i-k = 10 μm; h = 70 μm; l = 1 μm; m, n = 500 nm.

Unexpanded basidiomata 13–28 mm, cylindrical to ellipsoidal, very light grey (#fdfdfd) to very pale red (#ffe6e6) with a slight protrusion, rough. Expanded basidiomata height 35–70 mm, diameter 18–37 mm, deep saccate, **Exoperidium** splitting into 6, arched, not hygrometric, prosthecae length 23–35 mm, diameter 5–13 mm, exoperidium attached to the rhizomorphs. Rhizomorphs with 0.1–5.4 μm hyphae, fibrous and transparent, white (#ffffff). **Mycelial layer** 49.5–59.0 μm, consisting of transparent hyphae (1.0–3.5 μm) with thin walls and no septum, curved. **Fibrous layer** 6.5–16.5 μm, transparent, curved, thick-walled hyphae (1.1–5.0 μm) smooth, transparent to cream (#fffdd0), pure red (#e60000) to dark red (#9a0000) when stained with Congo red. **Pseudoparenchymatous layer** 2.5–19.3 × 2.7–30.4 μm, irregular shape, mycelium is transparent when fresh, pure orange (#ffa500) to moderate pink (#cc6691) when stained with Congo red, the thickness of the pseudoparenchyma layer is about 1.0–1.3 mm, very soft pink (#d98ca0). **Endoperidial body** 11–23 mm, globose, sessile, very light grey (#dfdfdf) to dark grey (#a0a0a0), with lighter reticulation. Endoperidial surface with some protruding hyphae, endoperidium is interwoven by transparent hyphae, fibrous. **Peristome** fibrillose, unpleated, wide conical, with obvious oral margin ring. Columella obvious very light grey (#f4f4f4 to #e0e0e0). **Eucapillitium hyphae** 1.0–5.5 μm, thick-walled, with distinct cavities, smooth, the ends tapering and are bluntly rounded (Fig. 1).

##### Basidiospores globose

Holotype (40/2/1) 4.5–5.3–6.0 × (4.5)5.0–5.4–6.0 μm, Q = (0.80)0.83–1.12(1.14), Qm= 0.98 ± 0.08, n = 40, including spines truncated at the apex ornamentation, with 0.2–0.5 µm high warts, ornamentation isolated or coalescing crest-like warts. Basidia not observed.

#### Diagnosis

*Geastrumsuae* is characterised by long stipes and larger basidiomata; Pseudoparenchymatous layer is pink, smooth; globose endoperidial body, grey; the ends of eucapillitium hyphae taper and are bluntly rounded; and they live in groups.

#### Etymology

The species is named suae (Lat.), in memory of the Chinese mycologist Prof. Hong-Yan Su, who kindly helped the authors in many ways and sadly passed away on 3 May 2022 during the preparation of the current paper.

#### Habit

It grows in groups on the ground in mixed coniferous and broad-leaved forests where there are *Alnusnepalensis* and *Pinusyunnanensis*, with thick humus. Currently, it is known only from Cangshan Mountain.

## Analysis

### Phylogenetic analysis

**Figure 2.**
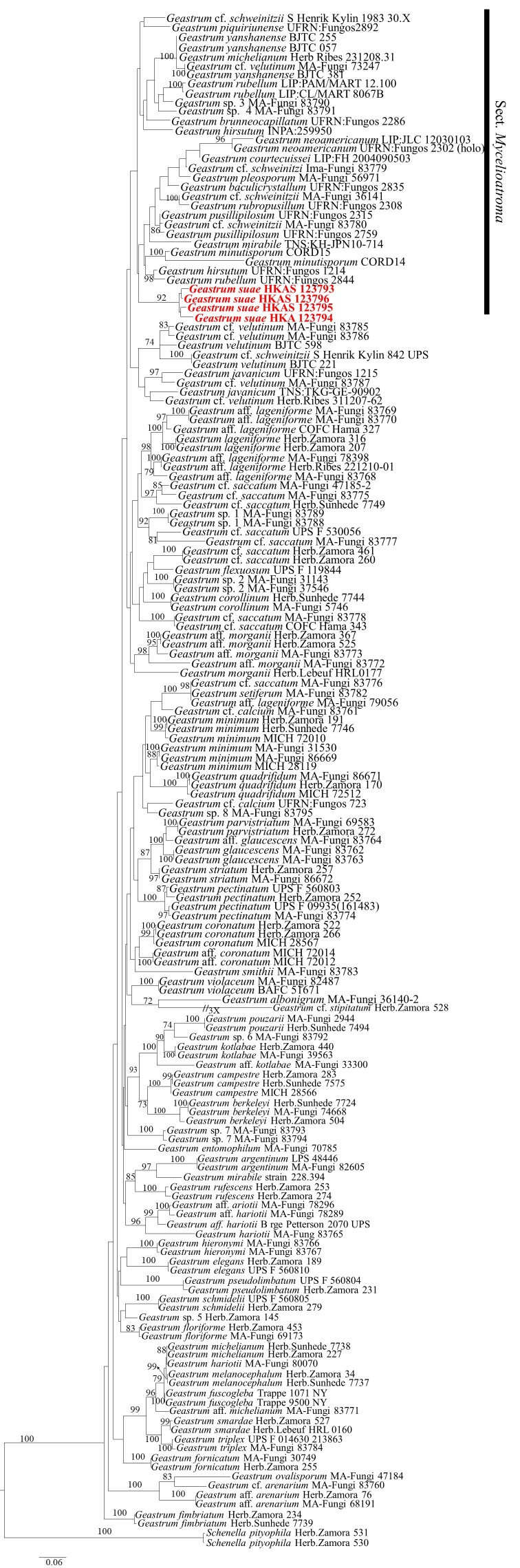
Phylogenetic tree of *Geastrum* species and related taxa, based on ITS and nrLSU sequence data.

**Figure 3.**
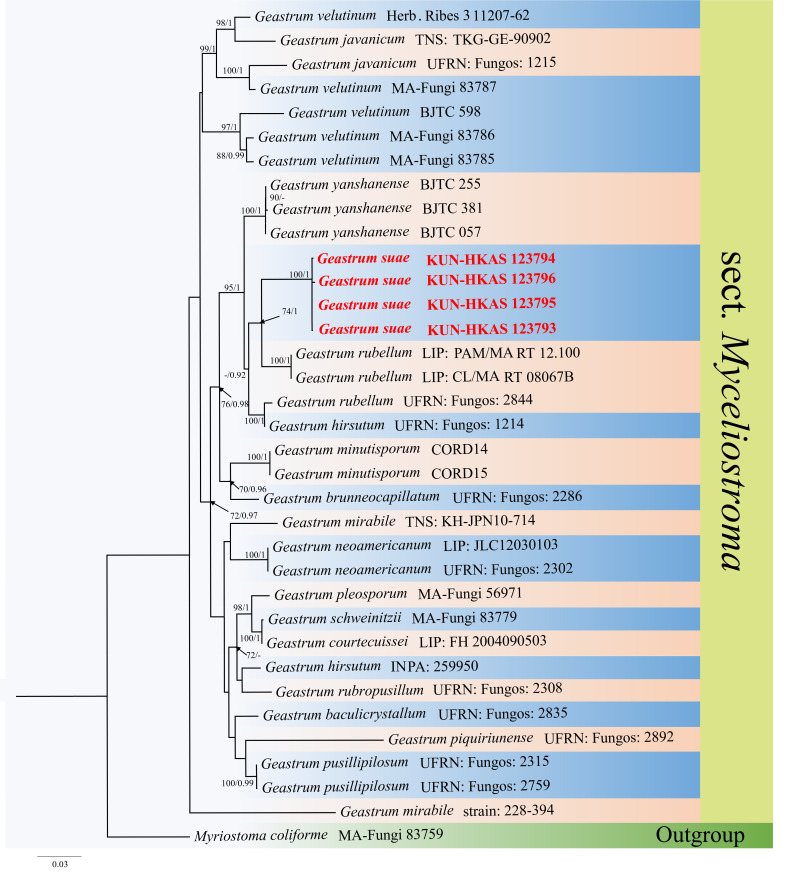
Phylogenetic tree of the new Geastrum species and related taxa which belong to sect. Myceliostroma, based on ITS and nrLSU sequence data. Branches are labelled with bootstrap values (ML) higher than 70% and posterior probabilities (PP) higher than 0.95. The new species are shown in red bold.

Firstly, we constructed the ML tree of *Geastrum* genus, based on ITS (1–540 bp) and nrLSU (541–1498 bp) genes and found that *G.suae* is in Sect. Mycelioatroma. The Maximum Likelihood bootstrap values (ML) equal to or greater than 70% are given above each node (Fig. 2), with the Final ML Optimisation Likelihood: -24127.230142. The aligned matrix had 856 distinct alignment patterns, with 6.78% completely undetermined characters or gaps. The base frequency and rate are as follows: A = 0.274187, C = 0.208839, G = 0.265219, T = 0.251755; rate AC = 1.202699, AG = 3.054698, AT = 1.472914, CG = 0.671195, CT = 5.726232, GT = 1.000000; gamma distribution shape: α = 0.269052. Therefore, we constructed the ML tree and Bayesian tree of Sect. Mycelioatroma, based on ITS and nrLSU genes and clarified the position of *G.suae* in this Section. The dataset is composed of ITS and nrLSU genes, comprising a total of 1478 characters including gaps, ITS (1–591 bp) and nrLSU (592–1478 bp), including 35 taxa with *Myriostomacoliforme* (MA-Fungi 83759) as the outgroup taxon (Fig. 3). The best fit model for the combined 2-gene dataset estimated and applied in the Bayesian analysis was GTR+I+G, lset nst = 6, rates = invgamma; prset statefreqpr = dirichlet (1,1,1,1). The phylogenetic analysis of ML and BI produces similar topology. The combined dataset analysis of RAxML generates a best-scoring tree (Fig. 3), with the Final ML Optimisation Likelihood value of -7513.207751. The aligned matrix had 584 distinct alignment patterns, with 21.33% completely undetermined characters or gaps. The base frequency and rate are as follows: A = 0.272494, C = 0.207593, G = 0.257821, T = 0.262093; rate AC = 1.093594, AG = 2.765430, AT = 1.755140, CG = 0.441983, CT = 5.721217, GT = 1.000000; gamma distribution shape: α = 0.243957. Bootstrap support values with ML greater than 70% and Bayesian posterior probabilities (PP) greater than 0.95 are given above the nodes (Fig. 3).

Phylogenetic analysis showed that four new collections of *G.suae* clustered together with high bootstrap support and are sister to *G.rubellum* with good bootstrap support (74% ML/1 PP Fig. 3).

## Discussion

*Geastrumsuae* can be easily recognised by the basidiomata with pink neat, smooth 6-lobed ectoderm, globose sessile endoperidium and longer prosthecae.

In the phylogenetic inferences, *Geastrumsuae* is sister to *G.rubellum*, which is known from the biome Tropical and Subtropical Moist Broadleaf Forests in Brazil (Accioly et al. 2019) (Fig. 3). Morphologically, both species share similar characteristics of the mesopodal basidiomata, but *G.rubellum* has reddish to brownish exoperidium with longer exoperidium hairs. *G.suae* hardly has such hairs and the reddish pseudoparenchymatous layer in *G.rubellum* also clearly differentiates *G.suae*. Not only that, but *G.rubellum* also has reddish to brownish exoperidium with a verrucose to hairy mycelial layer, while the exoperidium of *G.suae* is almost smooth. Their size is different, the expanded basidiomata saccate of *G.rubellum* being 10 mm high × 8.5–30 mm wide, while *G.suae* is 35-70 mm high × 18–37 mm wide. The warts on the basisiospore of *G.suae* are shorter than those of *G.rubellum*. The pseudoparenchymatous layer of *G.rubellum* is pure (or mostly pure) pink (#fa007d) when fresh, brownish-grey when dried, but is very pale red (#ffccd5) for *G.suae*. The ITS comparison between our specimen (KUN-HKAS 123795) and *G.rubellum* (LIP: PAM/MART 12.100) revealed a 53 bp difference in a total of 542 bp. The nrLSU comparison between *G.suae* (KUN-HKAS 123795) and *G.rubellum* (LIP: PAM/MART 12.100) revealed 11 bp difference in a total of 809 bp (Accioly et al. 2019). It is worth noting that *G.rubellum* is distributed in the Neotropics (Accioly et al. 2019). Combined with the above analysis, we introduce *Geastrumsuae* as a new species.

## Supplementary Material

XML Treatment for Geastrum suae

## Figures and Tables

**Table 1.** Amplification primers information used in this study.

Gene	Primer	Primer sequence (5ʹ-3ʹ)	References
ITS	ITS1F	CTTGGTCATTTAGAGGAAGTAA	Gardes and Bruns (1993)
	ITS4	TCCTCCGCTTATTGATATGC	White et al. (1990)
nrLSU	LR0R	ACCCGCTGAACTTAAGC	Vilgalys and Hester (1990)
	LR5	ATCCTGAGGGAAACTTC	Vilgalys and Hester (1990)

**Table 2.** Species, specimens, Collection locality and GenBank accession numbers of sequences used in this study (newly-generated sequences are indicated in bold).

**Species**	**Strain/Voucher**	**Collection locality**	**GenBank Accession No.**	
			**ITS**	**nrLSU**
*Geastrummirabile*	strain: 228-394	Japan	AB509736	-
*Geastrumjavanicum*	TNS:TKG-GE-90902	Japan	JN845100	JN845218
*Geastrummirabile*	TNS:KH-JPN10-714	Japan	JN845109	JN845227
*Geastrumparvistriatum*	MA-Fungi 69583	Spain	JN943160	JN939560
*Geastrumparvistriatum*	Herb. Zamora 272	Spain	JN943162	JN939572
*Geastrumstriatum*	Herb. Zamora 257	Spain	JN943164	JN939557
*Geastrumcampestre*	Herb. Zamora 283	Spain	JN943167	JN939575
Geastrumaff.arenarium	Herb. Zamora 76	Spain	KF988338	KF988470
*Geastrumlageniforme*	Herb. Zamora 316	Spain	KF988339	KF988514
Geastrumcf.calceum	UFRN-Fungos 723	Brazil	KF988340	KF988477
Geastrumcf.calceum	MA-Fungi 83761	Argentina	KF988341	KF988478
Geastrumaff.hariotii	Börge Petterson 2070	Mozambique	KF988342	KF988507
Geastrumcf.saccatum	Herb. Sunhede 7749	Australia	KF988343	KF988556
*Geastrumhieronymi*	MA-Fungi 83767	Argentina	KF988344	KF988509
Geastrumcf.stipitatum	Herb. Zamora 528	Brazil	KF988345	KF988576
*Geastrumalbonigrum*	MA-Fungi 36140-2	Panama	KF988349	KF988468
Geastrumaff.arenarium	MA-Fungi 68191	Spain	KF988350	KF988469
Geastrumcf.arenarium	MA-Fungi 83760	Argentina	KF988351	KF988471
*Geastrumargentinum*	LPS 48446	Argentina	KF988352	KF988472
*Geastrumargentinum*	MA-Fungi 82605	Argentina	KF988353	KF988473
*Geastrumberkeleyi*	MA-Fungi 74668	Spain	KF988354	KF988474
*Geastrumberkeleyi*	Herb. Sunhede 7724	Sweden	KF988355	KF988475
*Geastrumberkeleyi*	Herb. Zamora 504	Sweden	KF988356	KF988476
*Geastrumcampestre*	Herb. Sunhede 7575	Sweden	KF988357	KF988479
*Geastrumcampestre*	MICH 28566	USA	KF988358	KF988480
*Geastrumcorollinum*	MA-Fungi 5746	Spain	KF988359	KF988481
*Geastrumcorollinum*	Herb. Sunhede 7744	Sweden	KF988360	KF988482
*Geastrumcoronatum*	Herb. Zamora 266	Spain	KF988361	KF988483
*Geastrumcoronatum*	Herb. Zamora 522	Sweden	KF988362	KF988484
*Geastrumcoronatum*	MICH 28567	USA	KF988363	KF988485
Geastrumaff.coronatum	MICH 72012	USA	KF988364	KF988486
Geastrumaff.coronatum	MICH 72014	USA	KF988365	KF988487
*Geastrumelegans*	Herb. Zamora 189	Spain	KF988366	KF988488
*Geastrumelegans*	UPS F-560810	Sweden	KF988367	KF988489
*Geastrumentomophilum*	MA-Fungi 70785	Brazil	KF988368	KF988490
*Geastrumfimbriatum*	Herb. Zamora 234	Spain	KF988369	KF988491
*Geastrumfimbriatum*	Herb. Sunhede 7739	Sweden	KF988370	KF988492
*Geastrumflexuosum*	UPS F-119844	Sweden	KF988371	KF988493
*Geastrumfloriforme*	MA-Fungi 69173	Spain	KF988372	KF988494
*Geastrumfloriforme*	Herb. Zamora 453	Spain	KF988373	KF988495
*Geastrumfornicatum*	Herb. Zamora 255	Spain	KF988374	KF988496
*Geastrumfornicatum*	MA-Fungi 30749	Spain	KF988375	KF988497
*Geastrumfuscogleba*	NY Trappe 1071	USA	KF988376	KF988498
*Geastrumfuscogleba*	NY Trappe 9500	USA	KF988377	KF988499
*Geastrumglaucescens*	MA-Fungi 83762	Argentina	KF988378	KF988500
*Geastrumglaucescens*	MA-Fungi 83763	Argentina	KF988379	KF988501
Geastrumaff.glaucescens	MA-Fungi 83764	Argentina	KF988380	KF988502
*Geastrumhariotii*	MA-Fungi 83765	Argentina	KF988381	KF988504
Geastrumaff.hariotii	MA-Fungi 78296	Brazil	KF988382	KF988505
Geastrumaff.hariotii	MA-Fungi 78289	Brazil	KF988383	KF988506
*Geastrumhieronymi*	MA-Fungi 83766	Argentina	KF988384	KF988508
*Geastrumkotlabae*	MA-Fungi 39563	Spain	KF988385	KF988510
*Geastrumkotlabae*	Herb. Zamora 440	Spain	KF988386	KF988511
Geastrumaff.kotlabae	MA-Fungi 33300	Tanzania	KF988387	KF988512
*Geastrumlageniforme*	Herb. Zamora 207	Spain	KF988388	KF988513
Geastrumaff.lageniforme	MA-Fungi 83768	Argentina	KF988389	KF988516
Geastrumaff.lageniforme	COFC Hama 327	Niger	KF988390	KF988517
Geastrumaff.lageniforme	MA-Fungi 83770	Argentina	KF988391	KF988518
Geastrumaff.lageniforme	MA-Fungi 83769)	Argentina	KF988392	KF988519
Geastrumaff.lageniforme	MA-Fungi 78398	Portugal	KF988393	KF988520
Geastrumaff.lageniforme	Herb. Ribes 221210-01	Spain	KF988394	KF988521
*Geastrummelanocephalum*	Herb. Zamora 34	Spain	KF988395	KF988522
*Geastrummelanocephalum*	Herb. Sunhede 7737	Sweden	KF988396	KF988523
*Geastrummichelianum*	Herb. Sunhede 7738	Sweden	KF988397	KF988524
*Geastrummichelianum*	Herb. Zamora 227	Spain	KF988398	KF988525
Geastrumaff.michelianum	MA-Fungi 83771	Argentina	KF988399	KF988527
*Geastrumminimum*	Herb. Zamora 191	Spain	KF988400	KF988528
*Geastrumminimum*	Herb. Sunhede 7746	Sweden	KF988401	KF988529
*Geastrumminimum*	MICH 72010	USA	KF988402	KF988530
*Geastrumminimum*	MICH 28119	Spain	KF988403	KF988531
*Geastrumminimum*	MA-Fungi 31530	USA	KF988404	KF988532
*Geastrumminimum*	MA-Fungi 86669	Sweden	KF988405	KF988533
*Geastrummorganii*	Herb. Lebeuf HRL0177	Canada	KF988406	KF988534
Geastrumaff.morganii	Herb. Zamora 367	Spain	KF988407	KF988535
Geastrumaff.morganii	Herb. Zamora 525	Spain	KF988408	KF988536
Geastrumaff.morganii	MA-Fungi 83772	Argentina	KF988409	KF988537
Geastrumaff.morganii	MA-Fungi 83773	Argentina	KF988410	KF988538
*Geastrumovalisporum*	MA-Fungi 47184	Bolivia	KF988411	KF988539
*Geastrumpectinatum*	Herb. Zamora 252	Spain	KF988412	KF988540
*Geastrumpectinatum*	UPS F-560803	Sweden	KF988413	KF988541
*Geastrumpectinatum*	UPS F-09935 (161483)	Tanzania	KF988414	KF988542
*Geastrumpectinatum*	MA-Fungi 83774	Argentina	KF988415	KF988543
*Geastrumpleosporum*	MA-Fungi 56971	Cameroon	KF988416	KF988544
*Geastrumpouzarii*	MA-Fungi 2944	Czechoslovakia	KF988417	KF988545
*Geastrumpouzarii*	Herb. Sunhede 7494	Czechoslovakia	KF988418	KF988546
*Geastrumpseudolimbatum*	Herb. Zamora 231	Spain	KF988419	KF988547
*Geastrumpseudolimbatum*	UPS F-560804	Sweden	KF988420	KF988548
*Geastrumquadrifidum*	Herb. Zamora 170	Spain	KF988421	KF988549
*Geastrumquadrifidum*	MA-Fungi 86671	Sweden	KF988422	KF988550
*Geastrumquadrifidum*	MICH 72512	USA	KF988423	KF988551
*Geastrumrufescens*	Herb. Zamora 253	Spain	KF988424	KF988552
*Geastrumrufescens*	Herb. Zamora 274	Spain	KF988425	KF988553
Geastrumcf.saccatum	MA-Fungi 47185-2	Bolivia	KF988426	KF988554
Geastrumcf.saccatum	MA-Fungi 83775	Argentina	KF988427	KF988555
Geastrumcf.saccatum	UPS F-530056	Japan	KF988428	KF988558
Geastrumcf.saccatum	MA-Fungi 83777	Argentina	KF988429	KF988559
Geastrumcf.saccatum	Herb. Zamora 260	Spain	KF988430	KF988560
Geastrumcf.saccatum	Herb. Zamora 461	Spain	KF988431	KF988561
Geastrumcf.saccatum	COFC Hama 343	Niger	KF988432	KF988562
Geastrumcf.saccatum	MA-Fungi 83778	Argentina	KF988433	KF988563
*Geastrumschmidelii*	Herb. Zamora 279	Spain	KF988434	KF988564
*Geastrumschmidelii*	UPS F-560805	Sweden	KF988435	KF988565
Geastrumcf.schweinitzii	S Henrik Kylin 1983 30.X	Papua New Guinea	KF988436	KF988566
Geastrumcf.schweinitzii	MA-Fungi 83779	Argentina	KF988437	KF988567
Geastrumcf.schweinitzii	MA-Fungi 36141	Panama	KF988438	KF988568
Geastrumcf.schweinitzii	MA-Fungi 83780	Argentina	KF988439	KF988569
*Geastrumsmardae*	Herb. Lebeuf HRL 0160	Canada	KF988440	KF988573
*Geastrumsmardae*	Herb. Zamora 527	Spain	KF988441	KF988574
*Geastrumsmithii*	MA-Fungi 83783	Argentina	KF988442	KF988575
*Geastrumstriatum*	MA-Fungi 86672	Sweden	KF988443	KF988577
*Geastrum* “*triplex*”	UPS F-014630 (213863)	Madagascar	KF988444	KF988578
*Geastrum* “*triplex*”	MA-Fungi 83784	Argentina	KF988445	KF988579
Geastrumcf.velutinum	MA-Fungi 83785	Argentina	KF988446	KF988581
Geastrumcf.velutinum	MA-Fungi 83786	Argentina	KF988447	KF988582
Geastrumcf.velutinum	Herb. Ribes 311207-62	Spain	KF988448	KF988583
Geastrumcf.velutinum	MA-Fungi 83787	Peru	KF988449	KF988584
*Geastrumviolaceum*	BAFC 51671	Argentina	KF988450	KF988585
*Geastrumviolaceum*	MA-Fungi 82487	Argentina	KF988451	KF988586
*Geastrum* sp.1	MA-Fungi 83788	Argentina	KF988452	KF988587
*Geastrum* sp.1	MA-Fungi 83789	Argentina	KF988453	KF988588
*Geastrum* sp.2	MA-Fungi 31143	Spain	KF988454	KF988589
*Geastrum* sp.2	MA-Fungi 37546	Spain	KF988455	KF988590
*Geastrum* sp.3	MA-Fungi 83790	Argentina	KF988456	KF988591
*Geastrum* sp.4	MA-Fungi 83791	Peru	KF988457	KF988592
*Geastrum* sp.5	Herb. Zamora 145	Spain	KF988458	KF988593
*Geastrum* sp.5	Herb. Zamora 450	Spain	KF988459	KF988594
*Geastrum* sp.6	MA-Fungi 83792	Argentina	KF988460	KF988595
*Geastrum* sp.7	MA-Fungi 83793	Argentina	KF988461	KF988596
*Geastrum* sp.7	MA-Fungi 83794	Argentina	KF988462	KF988597
*Geastrum* sp.8	MA-Fungi 83795	Argentina	KF988463	KF988598
*Geastrumhirsutum*	UFRN-Fungos 1214	Brazil	KJ127029	-
*Geastrumjavanicum*	UFRN-Fungos 1215	Brazil	KJ127031	-
*Geastrumminutisporum*	CORD14	Argentina	KM260664	-
*Geastrumminutisporum*	CORD15	Argentina	KM260665	-
*Geastrumpusillipilosum*	UFRN:Fungos 2315	Brazil	KX761175	KX761176
*Geastrumpusillipilosum*	UFRN:Fungos 2759	Brazil	KX761177	KX761178
*Geastrumpiquiriunense*	UFRN:Fungos:2892	Brazil	MH260269	MH260270
*Geastrumhirsutum*	INPA:259950	Brazil	MH634993	MH635026
*Geastrumrubropusillum*	UFRN:Fungos:2308	Brazil	MH634994	MH635027
*Geastrumbaculicrystallum*	UFRN:Fungos:2835	Brazil	MH634995	MH635028
*Geastrumbrunneocapillatum*	UFRN:Fungos:2286	Brazil	MH634996	MH635029
*Geastrumrubellum*	UFRN:Fungos:2844	Brazil	MH634999	MH635031
*Geastrumneoamericanum*	UFRN:Fungos:2302	Brazil	MH635001	MH635040
*Geastrumcourtecuissei*	LIP:FH 2004090503	Guadeloupe	MH635003	MH635033
*Geastrumrubellum*	LIP:CL/MART 8067B	Martinique	MH635009	-
*Geastrumrubellum*	LIP:PAM/MART 12.100	Martinique	MH635010	MH635037
*Geastrumneoamericanum*	LIP:JLC 12030103	French	MH635014	MH635038
***Geastrumsuae***	**HKAS 123795 (Holotype)**	**China**	**ON529511**	**ON529515**
***Geastrumsuae***	**HKAS 123794**	**China**	**ON529512**	**ON529516**
***Geastrumsuae***	**HKAS 123793**	**China**	**ON529513**	**ON529517**
***Geastrumsuae***	**HKAS 123796 (Paratype**）	**China**	**ON529514**	**ON529518**
*Geastrumhariotii*	MA-Fungi 80070	Dominican Republic	-	KF988503
Geastrumaff.lageniforme	MA-Fungi 79056	Brazil	-	KF988515
Geastrumcf.saccatum	MA-Fungi 83776	Argentina	-	KF988557
Geastrumcf.schweinitzii	S Henrik Kylin 842	Fiji	-	KF988570
Geastrumcf.velutinum	MA-Fungi 73247	India	-	KF988580
*Geastrummichelianum*	Herb. Ribes 231208-31	Spain	-	KF988526
*Geastrumsetiferum*	MA-Fungi 83781	Argentina	-	KF988571
*Geastrumsetiferum*	MA-Fungi 83782	Argentina	-	KF988572
*Geastrumvelutinum*	BJTC 221	China	-	MZ509382
*Geastrumvelutinum*	BJTC 598	China	MZ508877	-
*Geastrumyanshanense*	BJTC 381	China	MZ508878	MZ509383
*Geastrumyanshanense*	BJTC 057	China	MZ508879	MZ509384
*Geastrumyanshanense*	BJTC 255	China	MZ508880	-
*Schenellapityophila*	Herb. Zamora 530	Spain	KF988346	KF988464
*Schenellapityophila*	Herb. Zamora 531	Spain	KF988347	KF988465
*Myriostomacoliforme*	MA-Fungi 83759	Argentina	KF988348	KF988467
